# Adapting and Validating a Scale to Measure Sexual Stigma among Lesbian, Bisexual and Queer Women

**DOI:** 10.1371/journal.pone.0116198

**Published:** 2015-02-13

**Authors:** Carmen H. Logie, Valerie Earnshaw

**Affiliations:** 1 Factor-Inwentash Faculty of Social Work, University of Toronto, Toronto, Canada; 2 Women’s College Research Institute, University of Toronto, Toronto, Canada; 3 Harvard Medical School, Harvard University, Boston, United States of America; University of Perugia, ITALY

## Abstract

Lesbian, bisexual and queer (LBQ) women experience pervasive sexual stigma that harms wellbeing. Stigma is a multi-dimensional construct and includes perceived stigma, awareness of negative attitudes towards one’s group, and enacted stigma, overt experiences of discrimination. Despite its complexity, sexual stigma research has generally explored singular forms of sexual stigma among LBQ women. The study objective was to develop a scale to assess perceived and enacted sexual stigma among LBQ women. We adapted a sexual stigma scale for use with LBQ women. The validation process involved 3 phases. First, we held a focus group where we engaged a purposively selected group of key informants in cognitive interviewing techniques to modify the survey items to enhance relevance to LBQ women. Second, we implemented an internet-based, cross-sectional survey with LBQ women (n=466) in Toronto, Canada. Third, we administered an internet-based survey at baseline and 6-week follow-up with LBQ women in Toronto (n=24) and Calgary (n=20). We conducted an exploratory factor analysis using principal components analysis and descriptive statistics to explore health and demographic correlates of the sexual stigma scale. Analyses yielded one scale with two factors: perceived and enacted sexual stigma. The total scale and subscales demonstrated adequate internal reliability (total scale alpha coefficient: 0.78; perceived sub-scale: 0.70; enacted sub-scale: 0.72), test-retest reliability, and construct validity. Perceived and enacted sexual stigma were associated with higher rates of depressive symptoms and lower self-esteem, social support, and self-rated health scores. Results suggest this sexual stigma scale adapted for LBQ women has good psychometric properties and addresses enacted and perceived stigma dimensions. The overwhelming majority of participants reported experiences of perceived sexual stigma. This underscores the importance of moving beyond a singular focus on discrimination to explore perceptions of social judgment, negative attitudes and social norms.

## Introduction

Lesbian, gay, bisexual, queer (LGBQ) and other sexually diverse persons experience widespread stigma and discrimination with deleterious impacts on wellbeing [1–4]. Sexual stigma refers to social and structural processes of devaluation, power inequities, and negative attitudes and stereotypes towards LGBQ persons, relationships and communities [5]. Conceptualizations of sexual stigma highlight processes of social and institutional exclusion of LGBQ persons; this builds on the more individualized focus of homophobia literature that refers to individuals’ fear, hostility and discrimination directed at LGBQ persons [6,7].

Stigma experienced by LGBQ persons is multi-dimensional. Perceived, or felt-normative, stigma includes one’s awareness of negative attitudes and treatment towards one’s group (e.g. LGBQ) and fears of experiencing this discrimination [5,8]. Enacted stigma refers to overt experiences of discrimination, including physical, verbal and sexual violence and hate crimes [5,8]. Chronic stressors associated with sexual stigma contribute to health disparities among LGBQ persons [9–12].

Despite the multi-dimensional nature of stigma, research assessing sexual stigma and its health effects has typically explored one dimension of sexual stigma. For instance, many studies have explored enacted stigma, including discrimination and hate crimes [9,10,13–17]. To the extent that different forms of sexual stigma may be related to different health outcomes, however, it is important to measure multiple forms of stigma. Diaz et al. [18] developed and validated the ‘Homophobia Scale’ to assess both enacted and perceived/felt-normative stigma based on sexual orientation, racism and poverty among Latino gay, bisexual and other men who have sex with men (MSM) in the U.S. This scale has been used in other studies with Latino MSM [19], including Latino MSM living with HIV [20] and was validated among Black MSM in the US [21] and MSM in China [22]. We did not find any studies that adapted or validated this measure of sexual stigma among lesbian, bisexual or queer (LBQ) or other groups of women who have sex with women. In the present study we adapted and validated Diaz et al.’s [18] homophobia scale to assess perceived/felt-normative and enacted sexual stigma among LBQ women in Toronto and Calgary, Canada.

### Sexual Stigma and Wellbeing among Lesbian, Bisexual and Queer Women

A large evidence base indicates that LGBQ persons experience higher rates of depression [2,23–26], anxiety [2,23,25,26], and sexually transmitted infections (STI) [1,23,27] in comparison with their heterosexual counterparts. The evolving theoretical body of literature on sexual stigma often builds on Goffman’s [28] discussion of stigma produced through social processes of othering and exclusion targeting various identities (e.g. sexuality, ethnicity, disability). Meyer’s [4,12] minority stress model focused on stigma’s psychological impacts and articulated that chronic stress caused by sexual stigma contributes to health disparities among LGBQ persons. Structural analyses illuminate systems of power inequities produced and institutionalized in community and social norms, law and policy, healthcare, education, employment and other systems [29–31].

Enacted stigma has been associated with substance use [10,11,17], suicidal ideation [10,14,32], emotional and psychological distress [9,13,16], mental health issues [33], reduced sexual satisfaction [33] and sexual risk practices [10,34] among lesbian and bisexual (LB) women. Perceived stigma was associated with increased physical symptoms and negative mood [35] and exposure to stress [31] among LB women.

### Measurement of Sexual Stigma Among Lesbian, Bisexual and Queer Women

Conceptualizations of sexuality and stigma are shaped by a multiplicity of factors, including socio-cultural context and intersections with other identities such as gender, socio-economic status, race and ethnicity, religion and rural/urban location [5,36,37]. The unique lived realities of LBQ women, and the diversity of experiences among LBQ women of various identities, highlight the importance of explicitly exploring sexual stigma among LBQ women. Despite the associations discussed above between sexual stigma and deleterious health outcomes among LBQ women, few studies have focused on adapting and validating sexual stigma scales for ethnically diverse LBQ women.

We found no enacted sexual stigma scales validated among LBQ women; measures of enacted stigma have used a single item [9,33], focused on neighborhood statistics [14] or assessed overt acts of discrimination [11,16]. Limited research has validated measures of perceived stigma, and these measures have used different scales. Meyer, Schwartz & Frost [31] used a 6-item measure adapted from Link’s [38] mental illness stigma scale to measured stigma based on multiple social categories (e.g. gender, race, sexual orientation). In another study a 10-item Stigma Consciousness Scale was used by Lewis, Derlega, Clarke, & Kuang [35] adapted from Pinel [39] who validated this one-factor perceived stigma scale among lesbians (n = 27) in the US.

We did not find a composite measure of sexual stigma that included both enacted and perceived/felt-normative stigma that was validated among LBQ women. As mentioned above, Diaz et al.’s [18] scale included enacted and felt-normative stigma dimensions and was validated among MSM in the US [18–21] and in China [22]. The aim of this project was to adapt and validate this sexual stigma scale among ethnically diverse LBQ women in Toronto and Calgary, Canada.

## Methods

To meet our study objective we evaluated the psychometric properties of the sexual stigma scale by conducting an exploratory factor analysis within samples of Canadian LBQ women. Specifically, we evaluated its structural validity, internal reliability, construct validity, and test-retest reliability. We further explored mean differences on the scale by participant characteristics to identify any sub-groups differences in reported experiences of stigma within this sample.

We report findings from 2 studies where we tested the reliability of the sexual stigma scale with LBQ women.

### Study Design and Population

The first study had 2 phases; Phase 1 involved a focus group with key informants and Phase 2 involved an internet-based, structured cross-sectional survey. The focus group was conducted in November 2011 and the survey from December 2011-January 2012. Participant inclusion criteria for Phase1/2 included adults 18 years and older who identified as women who identified as lesbian, bisexual, queer (LBQ) and other women who have sex with women who lived in the Greater Toronto Area, in Ontario, Canada. We obtained Research Ethics Board approval from Women’s College Hospital for Study 1, including Phases 1 and 2, at the University of Toronto, Toronto, Canada (2011–0036-E).

The second study, Phase 3 (March 2014-May 2014), was a multi-center non-randomized cohort pilot STI prevention study with LBQ women in 2 urban locations: Calgary and Toronto, Canada[40]. Participant inclusion criteria included adults 18 years and older who identified as women who identified as lesbian, bisexual, queer (LBQ) and other women who have sex with women who lived in the Greater Toronto Area, in Ontario, or the Greater Calgary Area, Alberta. We obtained Research Ethics Board Approval from the University of Toronto’s HIV Research Ethics Board (Protocol Reference # 29291) and the University of Calgary’s Research Ethics Board (REB13–114) for Study 2 (Phase 3). In each phase we recruited and employed peer research assistants (PRA) who identified as LBQ women of diverse ages, ethnicities and sexualities to conduct participant recruitment.

### Phase 1

**Procedure and Sample.** We conducted a focus group with key informants (n = 10) to pilot test survey measures for LBQ women in Toronto. We purposively selected participants to include 5 LBQ women involved with producing LBQ women’s social and community events in Toronto, and 5 LBQ women who were health and service providers (e.g. social workers, outreach workers, counselors) in Toronto. We implemented techniques from cognitive interviewing [41] for sexual stigma scale items; each participant was provided a copy of the sexual stigma scale we adapted for LBQ women (detailed in the following section) and asked to carefully read over each item. We asked participants to consider how they would phrase items in their own words, how difficult the question was, and suggestions on how we could modify the items to enhance relevance for LBQ women [41].

We digitally recorded and transcribed the focus group verbatim; in addition we asked participants to mark their feedback and suggested changes for scale items directly on the paper copy. The study team used thematic analysis techniques[42] to identify, analyze and report themes in the written and oral data, paying particular attention to feedback on each scale item that could be integrated to enhance clarity and relevance. Feedback regarding each scale item was assessed, and items revised to incorporate small changes suggested by the majority of participants. This feedback contributed to further development of the scale and survey implementation in Phase 2. Participants provided written consent and signed informed consent documents.

**Measures.** Focus groups explored the applicability, and clarity, of Diaz et al.’s [18] ‘Homophobia Scale’ for LBQ women. Although named a ‘homophobia’ scale, the concepts in Diaz et al.’s [18] scale reflect conceptualizations of perceived and enacted sexual stigma; it is possible that the term sexual stigma became more commonly used in LGBQ research after Herek’s [7] seminal article differentiating the term homophobia from sexual stigma processes.

**Results.** We specified that items in the sexual stigma scale pertained to lesbian, bisexual and queer women as the prior scale referred to gay men. In the perceived stigma subscale, for several items we removed the specification that the statement was experienced as a child. For instance, instead of the item “How often as a child did you hear gays grow old alone” we asked “How often have you heard that lesbian, bisexual and queer women grow old alone?” Other changes to the enacted stigma scale included: 1) asking about homophobic violence generally rather than asking about violence as a child and adult separately (i.e. How often have you been hit or beaten up for being lesbian, queer or bisexual?); 2) asking about homophobic sexual violence separately than physical violence (i.e. How often have you been sexually assaulted for being lesbian, queer or bisexual?); 3) adding a housing discrimination question (i.e. How often have you lost a place to live for being lesbian, queer or bisexual?); 4) adding a friend discrimination question (i.e. How often have you lost your straight friends because you are lesbian, queer or bisexual?) Participants responded to all items on a 4-point Likert-type scale including: never, once or twice, a few times, many times. The final *Sexual Stigma Scale Adapted for Lesbian*, *Bisexual and Queer Women* included 12 items, 5 in the perceived stigma sub-scale and 7 in the enacted stigma sub-scale.

### Phase 2

**Procedure and Sample.** In Phase 2 we implemented a cross-sectional internet-based survey with LBQ women; the survey included the sexual stigma scale adapted for LBQ women in Phase 1 as well as a range of health outcomes discussed below. We used modified peer-driven recruitment sampling strategies, where PRA each recruited 25 participants, and convenience sampling using word-of-mouth, social networks (e.g. list-serves) and venue based recruitment (e.g. LGBQ agencies). The objective was to recruit 425 participants to complete a 60-minute self-administered online survey. In total, 466 participants completed the survey (Table 1). Participants provided online written informed consent through checking a box to signify agreement with the study processes prior to beginning the survey for Phase 2; it was mandatory for participants to provide informed consent prior to beginning the survey.

**Table 1:** Demographic characteristics of lesbian, bisexual and queer women participants in an online survey (n = 466) and STI intervention (n = 44) in Toronto, Canada.

	Phase 2 Sample	Phase 3 Sample
	% (n) or Range, M (SD)	% (n) or Range, M (SD)
Age	18–70, 31.38 (8.12)	22–44, 28.69 (5.74)
Highest Education Level		
High School Degree or Less	5.2 (24)	6.8 (3)
Some College	30.3 (141)	29.5 (13)
Bachelor Degree	32.8 (153)	47.7 (21)
Graduate Degree	24.7 (115)	15.9 (7)
Nativity		
Canada	71.5 (333)	72.7 (32)
Other	28.5 (133)	27.3 (12)
Ethno-Racial Identity		
White/Caucasian	59.9 (279)	54.5 (24)
Black/African	12.0 (56)	34.1 (15)
Asian	4.1 (19)	4.5 (2)
South Asian	3.9 (18)	
Indigenous/Aboriginal	3.2 (15)	
Other	5.4 (30)	6.8 (3)
Sexual Orientation		
Queer	44.2 (206)	50.0 (22)
Lesbian	27.3 (127)	29.5 (13)
Bisexual	15.5 (72)	18.2 (8)
Gay	4.1 (19)	
Other	9.0 (42)	2.3 (1)

*Note*: May not total 100% due to missing data

**Measures.** Based on feedback from Phase 1, we adapted Diaz et al.’s [18] Homophobia Scale to assess both perceived and enacted sexual stigma for lesbian, bisexual and queer women. We used the Patient Health Questionnaire-2 to assess depressive symptomology [43]. We measured self-esteem with the Single-Item Self-Esteem Scale where participants rated the statement: “I have self-esteem” on a five-point Likert scale [44]. To measure overall health we used a single global self-rated health item implemented by the World Health Organization (WHO) [45]. The Multi-dimensional Scale of Perceived Social Support [46], which includes sub-scales to measure family, friends and significant other support, was used to assess social support.

**Results.** To establish the structure of the scale, we conducted an exploratory factor analysis using principal components analysis with varimax rotation using data from the Phase 2 sample (Table 2). The exploratory factor analysis yielded two factors, accounting for 45.0% of the variance in the scale. Initial eigenvalues were 3.76 for factor 1 and 1.64 for factor 2; rotated eigenvalues were 2.73 and 2.66 respectively. Factor 1 reflected perceived or felt-normative stigma (i.e., hearing or feeling social devaluation of queer, lesbian, and bisexual women) and was titled Perceived Sexual Stigma. Factor 2 reflected experiences of enacted stigma from others (i.e., being treated poorly or unfairly by others because one is queer, lesbian, and/or bisexual) and was titled Enacted Sexual Stigma. Two items loaded onto both factors (factor loadings ≥.40), including: “How often have you been made fun of or called names for being lesbian, queer, or bisexual” and “How often have you lost your straight friends because you are lesbian, queer, or bisexual.” Both items reflect experiences of discrimination from others, and were therefore classified with factor 2 as part of the Enacted Sexual Stigma Subscale.

**Table 2:** Items factor loadings, mean (standard deviations), and proportions for the sexual stigma scale adapted for lesbian, bisexual and queer women (phase 2 sample: n = 466).

Factors and Items	Factor 1	Factor 2	M (SD)	Ever experienced % (n)
Factor 1: Perceived Sexual Stigma			2.67 (0.70)	92.7 (432)
How often have you heard that lesbian, bisexual and queer women are not normal?	0.64	0.13	3.40 (0.80)	96.3 (418)
How often have you had to pretend that you are straight in order to be accepted?	0.67	-0.20	2.92 (1.05)	80.9 (377)
How often have you heard that lesbian, bisexual and queer women grow old alone?	0.65	0.11	2.38 (1.09)	66.5 (310)
How often have you felt your family was hurt and embarrassed because you are lesbian, queer or bisexual?	0.68	0.21	2.66 (1.09)	75.5 (352)
How often have you felt you had to stop associating with your family because you are lesbian, queer or bisexual?	0.64	0.22	2.00 (1.11)	17.6 (82)
Factor 2: Enacted Sexual Stigma			1.51 (0.40)	84.8 (395)
How often have you been hit or beaten up for being lesbian, queer or bisexual?	0.03	0.74	1.22 (0.56)	14.8 (69)
How often have you been harassed by the police for being lesbian, queer or bisexual??	0.14	0.68	1.20 (0.51)	14.2 (66)
How often have you lost a place to live for being lesbian, queer or bisexual?	0.08	0.58	1.14 (0.37)	12.0 (56)
How often have you lost a job or career opportunity for being lesbian, queer or bisexual?	0.24	0.62	1.31 (0.61)	22.5 (105)
How often have you been sexually assaulted for being lesbian, queer or bisexual?	0.04	0.64	1.15 (0.44)	11.6 (54)
How often have you been made fun of or called names for being lesbian, queer or bisexual?	0.42	0.48	2.67 (1.02)	77.7 (362)
How often have you lost your straight friends because you are lesbian, queer or bisexual?	0.55	0.40	1.88 (0.84)	58.4 (272)
Total Sexual Stigma			2.00 (0.45)	92.9 (433)

To determine internal reliability, we calculated the Cronbach’s alpha of the total scale and the two subscales using data from the Phase 2 sample. The Total Sexual Stigma Scale had a Cronbach’s alpha of 0.78. The Perceived Sexual Stigma Subscale had a Cronbach’s alpha of 0.70 and the Enacted Sexual Stigma Subscale had a Cronbach’s alpha of 0.72. The total scale and subscales therefore demonstrated adequate internal reliability. Additional analyses suggest that psychometric properties are similar for women who identify as queer (total: Cronbach’s alpha = 0.75; perceived: Cronbach’s alpha = 0.67; enacted: Cronbach’s alpha = 0.69), lesbian (total: Cronbach’s alpha = 0.80; perceived: Cronbach’s alpha = 0.74; enacted: Cronbach’s alpha = 0.78), and bisexual (total: Cronbach’s alpha = 0.81; perceived: Cronbach’s alpha = 0.71; enacted: Cronbach’s alpha = 0.75).

To establish construct validity, we examined the correlations between the total scale and its subscales with indicators of mental and physical health using data from the Phase 2 sample (Table 3). All correlations were in the expected directions, indicating that women who scored higher on perceived and enacted sexual stigma also scored lower on indicators of mental and physical health. More specifically, women who scored higher on perceived and enacted sexual stigma also scored higher on depressive symptoms, lower on self-esteem, lower on social support from their families, and lower on overall health. Notably, the correlations between perceived and enacted sexual stigma with health outcomes differed in strength, further supporting the importance of differentiating between these two experiences of stigma. These results were similar across lesbian, bisexual and queer identified participants.

**Table 3:** Bivariate correlations between sexual stigma scale items, social support and health outcomes (phase 2 sample: n = 466).

	Total Sexual Stigma	Perceived Sexual Stigma	Enacted Sexual Stigma
Perceived Sexual Stigma	0.89**	1	
Enacted Sexual Stigma	0.82**	0.46**	1
Depression	0.25**	0.25**	0.17**
Self-esteem	-0.21**	-0.22**	-0.12*
Social Support: Family	-0.39**	-0.40**	-0.25**
Self-rated Health	-0.21**	-0.16**	-0.20**

*Note*: **p<.01;

*p<.05

We explored mean differences in scores on the Total Sexual Stigma Scale as well as the Perceived and Enacted Sexual Stigma Subscales by participant characteristics to identify any sub-groups that reported greater experiences of stigma using data from the Phase 2 sample (Table 4). There were no differences in sexual stigma scores by level of education. Women born outside of Canada scored higher on the Perceived Sexual Stigma Subscale, but there were no nativity differences on the Enacted Sexual Stigma Subscale or the Total Sexual Stigma Scale. Indigenous/Aboriginal and South Asian women tended to score highest, White/Caucasian and “other” women scored lowest, and African, Caribbean/Black and Asian women scored in between on the Total Sexual Stigma Scale and its subscales. Women who identified as queer scored the highest, women who identified as gay scored the lowest, and women who identified as lesbian, bisexual, or other scored in between on the total sexual stigma scale and the enacted sexual stigma subscale. They scored similarly on the perceived sexual stigma subscale. Additionally, there were no age differences on the Perceived Sexual Stigma Subscale or the Total Sexual Stigma Scale. Further, age was not correlated with the Total Sexual Stigma Scale or its subscales (all *p*s>.10).

**Table 4:** Characteristics associated with total sexual stigma, perceived sexual stigma and enacted sexual stigma mean differences, with higher scores indicating higher sexual stigma (Study 1, phase 2 survey sample: n = 466).

	Total Sexual Stigma	Perceived Sexual Stigma	Enacted Sexual Stigma
*Demographics*			
Highest Education Level	F(3,419) = 1.60, p = .19	F(3,419) = 1.86, p = .14	F(3,419) = 0.92, p = .43
High School Degree or Less	1.97 (0.51) _a_	2.63 (0.85) _a_	1.51 (0.40) _a_
Some College	1.94 (0.44) _a_	2.56 (0.72) _a_	1.49 (0.39) _a_
Bachelor Degree	2.00 (0.41) _a_	2.72 (0.66) _a_	1.49 (0.37) _a_
Graduate Degree	2.06 (0.39) _a_	2.75 (0.69) _a_	1.57 (0.46) _a_
Nativity	F(1,433) = 1.96, p = .16	F(1,433) = 4.74, p = .03	F(1,433) = 0.00, p = .99
Canada	1.98 (0.45) _a_	2.63 (0.70) _a_	1.51 (0.40) _a_
Other	2.05 (0.45) _a_	2.80 (0.70) _b_	1.51 (0.41) _a_
Ethno-Racial Identity	F(5,401) = 5.45, p<.001	F(5,401) = 3.32, p<.01	F(5,401) = 6.95, p<.001
White/Caucasian	1.95 (0.41) _a_	2.59 (0.68) _a_	1.48 (0.35) _a_
Black/African	2.00 (0.44) _a,b_	2.81 (0.70) _a,b_	1.43 (0.37) _a_
Asian	2.07 (0.49) _a,b_	2.80 (0.73) _a,b_	1.56 (0.47) _a,b_
South Asian	2.35 (0.50) _b_	3.13 (0.60) _b_	1.79 (0.63) _b,c_
Indigenous/Aboriginal	2.38 (0.50) _b_	2.94 (0.55) _a,b_	1.98 (0.57) _c_
Other	1.89 (0.48) _a_	2.56 (0.77) _a,b_	1.41 (0.38) _a_
Sexual Orientation	F(4,433) = 6.72, p<.001	F(4,433) = 2.39, p = .05	F(4.433) = 4.17, p<.01
Queer	2.06 (0.42) _a_	2.74 (0.67) _a_	1.58 (0.39) _a_
Lesbian	2.01 (0.46) _a,b_	2.70 (0.71) _a_	1.52 (0.44) _a,b_
Gay	1.72 (0.33) _b_	2.31 (0.65) _a_	1.31 (0.22) _b_
Bisexual	1.88 (0.47) _b_	2.56 (0.72) _a_	1.40 (0.39) _b_
Other	1.92 (0.52) _a,b_	2.60 (0.86) _a_	1.43 (0.37) _a,b_

*Note*: Means in the same column that share a subscript (a, b, c) are not significantly different at *p*≤0.05. Posthoc comparisons conducted using Bonferroni tests.

### Phase 3

**Procedure and Sample.** We pilot tested an HIV/STI prevention study with LBQ women in Calgary and Toronto, Canada [40]. Using modified peer-driven recruitment techniques LBQ women were recruited in Toronto (n = 24) and Calgary (n = 20) for a 2-day psycho-educational group-based HIV/STI prevention intervention. Participants completed a 60-minute self-administered online survey at baseline (directly before the intervention) and 6 weeks post-intervention. Participants provided online written informed consent through checking a box to signify agreement with the study processes prior to beginning the survey; providing online informed consent was mandatory for completing the surveys. Online informed consent was documented on the online survey database. These consent processes were approved by the research ethics boards.

**Measures.** We used the same sexual stigma measure described in Study 1, an adaptation of Diaz et al.’s [18] Homophobia Scale, to assess perceived and enacted sexual stigma among lesbian, bisexual and queer women.

**Results: Test-Retest Reliability.** To establish test-retest reliability, we examined correlations between scores on the Total Sexual Stigma Scale as well as the Perceived and Enacted Sexual Stigma Subscales over a 6-week interval. Scores on the Total Sexual Stigma Scale (*r* = 0.83, *p*<.001), Perceived Sexual Stigma Subscale (*r* = 0.70, *p*<.001), and Enacted Sexual Stigma Scale (*r* = 0.85, *p*<.001) were all correlated at large effect sizes, indicating strong test-retest reliability. The test-retest reliability of the Total, Perceived and Enacted Sexual Stigma Scale scores replicated across sub-groups of queer (n = 20) and lesbian (n = 12) participants; the sample size was too small (n = 8) to test correlations among bisexual participants.

## Discussion

Our study proposes a sexual stigma scale for LBQ women with good psychometric properties and two dimensions: enacted and perceived sexual stigma. For some of the items, particularly those in the enacted stigma sub-scale, most women reported rarely or never experiencing the event. Most of these items measured major or extreme forms of discrimination, such as being sexually assaulted, and therefore were not expected to happen frequently to participants in this sample. Despite their relative infrequency, they are important to study given their importance for wellbeing. The overwhelming majority of participants, however, did report experiencing perceived sexual stigma. This underscores the importance of conceptualizing and measuring the multi-dimensional components of sexual stigma and moving beyond a singular focus on discrimination and enacted stigma to also explore perceptions and fears of social judgment, negative attitudes and social norms captured in assessments of perceived stigma [5,8,31].

Sexual stigma experiences varied among LBQ women. Women who were Indigenous/Aboriginal and South Asian, and/or identify as queer scored higher on the sexual stigma scales and therefore may be particularly at risk of experiencing sexual stigma in Toronto. Our finding that Indigenous/Aboriginal and South Asian women experienced higher stigma is corroborated by prior research that highlights higher rates of sexual stigma among LGBQ persons of color than white persons [31]. Rusch et al. [47] also found higher rates of STI stigma among Aboriginal women in Vancouver than among non-Aboriginal women; their thoughtful discussion situated this finding of higher stigma in the context of cultural stigma and social exclusion. Prior research has highlighted higher rates of suicide among Black and Latino LGB youth than white LGB youth in the US [48], and higher rates of substance use among LGB persons who experience multiple forms of discrimination (racism, sexism, sexual stigma) [11]. Qualitative research with Aboriginal sexually diverse persons in Canada [49], and LBQ women of color in Toronto [50], revealed experiences of stigma and discrimination based on ethno-racial and sexual identities from both LGBQ communities and the larger society that contributed to stress and social exclusion. Understanding the intersection of sexuality and ethno-racial identity are key for understanding social and structural contexts of health among diverse LBQ women.

We did not locate other studies that explored differences in sexual stigma based on a queer identity; Kuyper et al.’s study in the Netherlands revealed lesbians reported more enacted stigma than bisexual women, but bisexuals had higher rates of internalized stigma [33]. Herek [51] highlighted higher enacted stigma among lesbian/gay persons than bisexuals in the US. Queer identities, grounded in the conceptualization of sexuality and gender as fluid, have often represented a socio-political identity that moves beyond the dichotomies of lesbian/heterosexual and being attracted to men/women [52]. It is possible that queer women experience higher rates of sexual stigma because they may not have as strong social support and positive group identity as those identifying as lesbian [53]. The phenomenon of queer women experiencing higher sexual stigma warrants further attention.

There is value in having a common measure, such as the Sexual Stigma Scale, to study these distinct experiences of sexual stigma among women with different sexual orientations (i.e. lesbian, bisexual, queer) and ethno-racial identities. Van Brakel [54] explained that there is much similarity in stigma experiences across diseases, and argued that using the same scale to measure stigma associated with different diseases can aid in comparing experiences of stigma and build theory to better understand disease stigma. Similarly, our intersectionality lens [36] allows us to build overarching understandings of sexual stigma while simultaneously attending to how experiences of stigma are influenced by, and intersect with, other social and sexual identities.

### Limitations and Future Directions

There are several limitations of our study. The sample size is small and therefore caution should be taken in interpreting results, particularly regarding ethno-racial differences in sexual stigma where there were small cell sizes. We recommend future studies with this scale include larger samples of ethno-racially and sexually diverse women. Stigma is a social construct, and the ways in which it is perceived and enacted may differ between social contexts [5,37]. Future studies should examine the extent to which the sexual stigma scale and its subscales are valid in other samples of LBQ women, including those from diverse sociocultural contexts.

While our scale addressed two dimensions of sexual stigma (perceived, enacted) we did not explore internalized stigma. Internalized sexual stigma refers to shame and lower self-worth among LGBQ persons associated with negative social attitudes towards this population [4,12]. Internalized stigma among LB women was associated with mental health problems [13,33,55–61], relationship issues [62], reduced sexual satisfaction [33], and lower self-esteem [56,63]. A measure of internalized stigma among LB women was validated in the US [63]. Future research with LBQ women could explore associations between internalized, perceived and enacted sexual stigma, assess relationships between different health outcomes and sexual stigma dimensions, and work towards a composite measure of these multiple dimensions of sexual stigma.

Despite these limitations, our study has several strengths. Our findings highlight the importance of an intersectional theoretical approach that looks at differences in experiences of sexual stigma among LBQ women, including the distinct experiences at the intersection of sexual identity and ethno-racial identities [63–65]. Future studies could measure intersectional forms of stigma experienced by LBQ women, including racism and sexism, to better understand the associations between sexual stigma and other forms of marginalization. This study represents the first psychometric evaluation of a multi-dimensional sexual stigma scale among LBQ women. We highlight the importance of addressing perceived stigma—social norms, values and expectations widely experienced among sexually diverse women in Calgary and Toronto—in addition to enacted stigma. Given that stigma toward LBQ women is pervasive and has negative health effects, it is critical to measure sexual stigma among LBQ women using psychometrically valid measures.
